# Context Matters: Divergent Roles of Exercise-Induced and Tumor-Derived Lactate in Cancer

**DOI:** 10.3390/biom15071010

**Published:** 2025-07-14

**Authors:** Amir hossein Ahmadi Hekmatikar, Ghazal Zolfaghari, Aref Basereh, D. Maryama Awang Daud, Kayvan Khoramipour

**Affiliations:** 1 Department of Physical Education and Sport Sciences, Faculty of Humanities, Tarbiat Modares University, Tehran 10600, Iran; a.hekmatikar4@gmail.com; 2 Department of Community Nutrition, Faculty of Medical Sciences and Technologies, Science and Research Branch, Islamic Azad University, Tehran 35131-19111, Iran; gszolfaghari@yahoo.com; 3 Department of Exercise Physiology, Faculty of Physical Education and Sport Science, Kharazmi University, Tehran 10600, Iran; basereh.aref@gmail.com; 4 Health Through Exercise and Active Living (HEAL) Research Unit, Faculty of Medicine and Health Sciences, Universiti Malaysia Sabah, Kota Kinabalu 88400, Sabah, Malaysia; 5 Department of Biomedical Sciences, Faculty of Medicine and Health Sciences, University Malaysia Sabah, Jalan UMS, Kota Kinabalu 88450, Sabah, Malaysia; 6 i+HeALTH Strategic Research Group, Department of Health Sciences, Miguel de Cervantes European University (UEMC), 47012 Valladolid, Spain

**Keywords:** lactate, exercise, cancer, metabolism

## Abstract

Instead of being waste product of metabolism, lactate, has become a key metabolic and signaling molecule in both exercise physiology and tumor biology. Carcinogenic cells produce huge amounts of lactate through the Warburg effect, which is a hallmark of aggressive tumors, increasing acidity in the environment that can stimulates angiogenesis, immune evasion, and metastasis. Conversely, while exercise acutely elevates blood lactate concentration but it consider helpful for cancer patients. This paradox raises the following question: is exercise-induced lactate a friend or foe in cancer? This study reviews current evidence on the mechanistic, metabolic, immunological, and clinical impacts of exercise-induced lactate in cancer patients, highlighting the context-dependent effects that render lactate either beneficial or detrimental. Tumor-derived lactate seems to be pro-tumorigenic, driving immune suppression and disease progression, whereas short bursts of lactate from exercise can enhance anti-tumor immunity and metabolic reprogramming under the right conditions. Therefore, lactate’s impact on cancer is “all about the context”.

## 1. Introduction

Lactate is a central metabolite at the crossroads of exercise physiology and cancer metabolism. During intense exercise, skeletal muscles switch to glycolytic metabolism, producing lactate as a byproduct to rapidly meet energy demands [1]. Historically, lactate was considered a “dead-end” waste metabolite; however, research now recognizes lactate as an important fuel and signaling molecule in the body [2]. In parallel, most cancer cells preferentially metabolize glucose via aerobic glycolysis (i.e., the Warburg effect), resulting in a high lactate output even in the presence of oxygen [3]. A phenomenon known as the “reverse Warburg effect” has also been described, in which cancer-associated stromal cells such as fibroblasts undergo aerobic glycolysis and secrete lactate, which is then taken up and utilized by adjacent cancer cells as a metabolic fuel [4]. This metabolic reprogramming confers growth advantages to tumors but also creates an acidic, lactate-rich tumor microenvironment (TME) [3]. Intriguingly, lactate accumulation has dual and seemingly contradictory implications: it is implicated in cancer progression, yet exercise—which transiently elevates lactate—is associated with reduced cancer risk and improved outcomes [2].

Regular physical activity is strongly correlated with a lower incidence of various cancers and better prognosis in cancer patients due to multifactorial benefits on body composition, hormone levels, immune function, and metabolism [5]. Clinical and epidemiological studies consistently show that patients with various types of cancer who engage in exercise programs (various protocols of resistance, endurance, and combined exercise) tolerate treatments better and may have reduced recurrence rates [6]. These benefits are often attributed to improved immune surveillance and metabolic health [7]. Nonetheless, the acute metabolic response to vigorous exercise (i.e., intensity higher than 70% of the maximal heart rate) includes spikes in blood lactate concentration [8]. This raises the following critical question: could exercise-induced lactate inadvertently “feed” cancer cells or worsen the TME, thereby acting as a foe? Some hypotheses, drawing on tumor metabolism parallels, suggest that high lactate could support tumor growth [8]. However, many studies showed positive effects for high-intensity training in various types of cancer, including breast [9], colorectal, prostate, lung, hematological, and gastrointestinal [10]. This discrepancy highlights that lactate’s role in cancer is highly context-dependent.

Recent advances in immunology and metabolism have begun to unravel this paradox. It appears that lactate can be both “friend” and “foe” to cancer patients: as a **friend**, by bolstering certain anti-tumor immune responses and adaptive metabolism [11], or as a **foe**, by facilitating tumor immune evasion and progression [12]. Key factors determining these outcomes include the concentration and duration of lactate exposure, the presence or absence of a buffering environment, tumor vs. systemic location of lactate, and interactions with specific cell types [13]. In essence, exercise-induced lactate operates in a physiological context profoundly different from tumor-generated lactate [14,15].

This review discusses (1) the mechanistic and metabolic underpinnings of lactate production in exercise and cancer; (2) the immunological effects of lactate, contrasting its immunosuppressive actions in the acidic TME with emerging evidence of immunostimulatory effects under certain conditions; (3) clinical implications for exercise in cancer patients and interventions targeting lactate metabolism; and (4) future directions for leveraging the context-dependent nature of lactate to improve cancer therapy. Understanding when lactate is a friend or foe will inform safer and more effective exercise prescriptions and metabolic treatments for cancer patients.

## 2. Mechanistic and Metabolic Perspectives of Lactate in Cancer vs. Exercise

**The Warburg Effect and Tumor Lactate Production:** Cancer cells commonly upregulate glycolysis and lactate production, even under aerobic conditions. This phenomenon results in tumors secreting high levels of lactate into the surrounding tissue. Although the Warburg effect is present in almost all cancer cells, the degree of dependency varies among cancer types. Breast cancer, glioblastoma, colorectal cancer, non-small cell lung cancer (NSCLC), and melanoma exhibit a high dependency on the Warburg effect. In contrast, renal cell carcinoma and chronic lymphocytic leukemia show a low dependency. Prostate cancer demonstrates low to moderate dependency, while thyroid cancer displays variable dependency on the Warburg effect [16].

Lactate is not merely a byproduct but appears to be integral to cancer’s survival strategy. San-Millán and Brooks [17] proposed that “dysregulated lactate metabolism and signaling are the key elements in carcinogenesis”, suggesting that the purpose of the Warburg effect is to enable excessive lactate generation. In their view, lactate is the one metabolic compound indispensable for all major hallmarks of cancer progression, including angiogenesis, immune escape, cell migration, metastasis, and self-sufficient metabolism. Tumor lactate production begins with oncogenes driving increased glucose uptake and glycolytic flux. Key mutations (e.g., in HIF-1, MYC [18], or p53 pathways [19]) rewire metabolism toward “lactagenesis”, a high glycolytic throughput with lactate accumulation and export. Breast, colorectal, lung, and glioblastoma cancers have been shown to exhibit a high expression of monocarboxylate transporters, particularly MCT1 and MCT4, although expression levels may vary by tumor subtype and microenvironmental factor [20]. The overexpression of these monocarboxylate shuttles can facilitate lactate transport: MCT4 on hypoxic/glycolytic cancer cells exports lactate [18,21], while MCT1 on better-oxygenated cancer cells and stromal cells imports lactate as a fuel [21,22]. This lactate shuttle within tumors creates metabolic symbiosis as lactate becomes an energy source for oxidative tumor cells and an intercellular signaling molecule [2]. This lactate shuttle could also be a therapeutic target: Benjamin et al. demonstrated, in highly glycolytic cancer cells, that the simultaneous pharmacological blockade of MCT1 and MCT4 traps lactate intracellularly and, when combined with metformin, leads to NAD⁺ depletion and selective tumor cell death [23].

**Lactate as a Signaling Molecule:** Far from a waste product, lactate can modulate numerous pathways. Lactate can bind to a G-protein coupled receptor, GPR81 (also known as HCAR1), in various cells, which inhibits adenylyl cyclase and can modulate immune and metabolic signals [24]. Lactate can also stabilize a hypoxia-inducible factor (HIF-1α), especially in endothelial cells, thus promoting angiogenesis. For instance, lactate was shown to activate HIF-1α and VEGF production in normoxic conditions, stimulating new blood vessel formation to supply growing tumors. Additionally, lactate serves as a precursor for histone lactylation, a recently discovered epigenetic mark [25]. In 2019, Zhang et al. demonstrated that lactate can directly modify histone lysine residues (adding a lactyl group), altering the gene expression in macrophages and other cells. This means that high lactate levels can reprogram cells at the epigenetic level, for example, driving tumor-associated macrophages (TAMs) toward a pro-tumor M2 phenotype by inducing genes like *ARG1* and *VEGF* that support tissue remodeling and immunosuppression. Lactate-fueled histone lactylation, therefore, links metabolism to gene expression in the TME [26].

**Metabolic Fate of Lactate—Tumor vs. Exercise Context:** In normal physiology, lactate is valuable metabolic currency. During acute exercise, muscles produce lactate, which is then systemically circulated. Well-trained individuals enhance lactate transport and oxidation; organs like the heart, liver, and even resting muscle fibers efficiently take up lactate and use it for ATP production or gluconeogenesis (the “lactate shuttle”) [27,28]. The presence of lactate normally signals cells to ramp up mitochondrial respiration to utilize it [29]. This acute response is accumulated as an adaptive response seen in chronic exercise training: repeated lactate exposure leads to increased mitochondrial density and MCT upregulation in muscles, improving lactate clearance and aerobic capacity [28]. In contrast, cancer cells often exhibit mitochondrial dysfunction or choose not to fully utilize oxidative pathways, despite lactate presence [27]. One hypothesis is that cancer cells avoid metabolizing lactate to keep the local concentration high because lactate may be more valuable as a signaling molecule to promote malignancy [27,30]. Studies suggest that if cancer cells are forced to oxidize lactate, their glycolytic drive and proliferation can diminish. Acidic lactate accumulation might, therefore, be a deliberate strategy by tumors to poison the local immune environment (as discussed in the next section) and to maintain an evolutionary advantage [15,30].

**Lactate, pH, and the Microenvironment:** It is important to distinguish between lactate (the anion, usually accompanied by a proton as lactic acid) and the resultant acidity. In tumors, lactate build-up often correlates with a lowered pH (lactic acid accumulation). Many pro-tumor effects traditionally ascribed to “lactate” are actually due to the low pH it creates [3]. The acidic TME can degrade the extracellular matrix, promote invasive behavior, and reduce the efficacy of certain therapies [31,32]. Moreover, acidosis drives the selection of more malignant cell clones [32,33]. However, recent work indicates that lactate itself (independent of H+) has signaling roles. For example, lactate can paradoxically act as a negative feedback regulator of glycolysis: high extracellular lactate can signal cancer cells to reduce their own glycolysis rate, likely to prevent over-acidification. This nuanced behavior underscores why context matters. The effect of lactate is concentration-dependent and cell-type-dependent [3,34,35].

**Exercise Intensity and Lactate Dynamics:** Exercise provides a useful physiological context to understand lactate’s dual nature. Mild to moderate aerobic exercise, 45 to 70% of the heart rate maximum, primarily uses oxidative metabolism, producing relatively little lactate and improving tissue oxygenation [36]. In contrast, high-intensity or anaerobic exercise systemically generates a surge of lactate, albeit transiently [37]. Notably, even very high lactate levels from exercise are acute (returning to the baseline within minutes to hours) and occur in a well-buffered systemic circulation [38]. Tumor-derived lactate, on the other hand, tends to be chronic and localized, with poor perfusion, leading to pockets of acidosis [3,39]. Thus, a burst of lactate during exercise is handled differently from a continuous lactate output by tumor cells in an inadequate lymphatic drainage context [40]. This distinction likely explains why exercise-induced lactate does not exhibit the same pathological effects; the body’s homeostatic mechanisms (increased blood flow, buffer systems, and liver clearance via the Cori cycle) rapidly mitigate lactate and prevent sustained acidity [35].

In summary, mechanistically, lactate sits at the intersection of cancer and exercise metabolism as a Janus-faced metabolite. In cancer, unrestrained “lactagenesis” supports tumor growth and survival through multiple avenues (metabolic, signaling, and micro environmental). In exercise, lactate production is a normal, regulated response that the body is equipped to utilize and clear. Table 1 provides the absolute concentration of lactate for cancer patients in different contexts. Warburg-driven lactate in tumors acts as a foe by sculpting a tumor-favorable niche. The same molecule, when produced by exercise in a controlled manner, can be a friend by serving as a fuel for healthy cells and possibly signaling beneficial adaptations. The following sections delve into how these mechanistic differences manifest in opposing effects on the immune system and clinical outcomes.

## 3. Immunological Effects of Lactate: Suppression in the TME vs. Activation with Exercise

**Lactate as an Immunosuppressant in the Tumor Microenvironment:** One of the most detrimental aspects of excess lactate in cancer is its ability to subvert anti-tumor immunity. High local lactate concentrations in the TME correlate with impaired function in immune cells tasked with attacking the tumor. Pioneering studies showed that lactic acid at levels found in tumors can severely blunt human T cell activity [46]. Fischer et al. reported that lactate accumulation suppressed cytotoxic T lymphocyte (CTL) proliferation and cytokine production by up to 95% and reduced their tumor-killing capacity, mainly by preventing T cells from exporting their own lactic acid. When T cells cannot efflux lactate (due to a reversed gradient across their membrane), intracellular acid builds up, deranging their metabolism and causing a loss of function. This mechanism explains why T cells infiltrating a highly glycolytic tumor become “paralyzed”—they are essentially swimming in the tumor’s lactic acid and cannot maintain their pH balance or bioenergetics [47]. Similarly, natural killer (NK) cells, which rely on perforin and granzyme exocytosis to kill tumor cells, are inhibited by lactate-induced acidosis. In highly glycolytic tumors, NK cells show decreased expressions of granzyme B and perforin, key molecules for cytotoxicity [48,49]. Brand et al. demonstrated that tumor-derived lactic acid selectively disables both T and NK cell activation, enabling cancer cells to escape immune surveillance [50].

Lactate in the TME also skews the composition of immune infiltrates toward immunosuppressive phenotypes. It promotes the polarization of macrophages to the M2-like TAM state at the expense of pro-inflammatory M1 macrophages [51]. TAMs exposed to lactate upregulate Arginase 1 and VEGF, secreting factors that suppress T cells and stimulate blood vessel growth, respectively [52]. This lactate-driven TAM polarization involves histone lactylation and the activation of the ERK/STAT3 pathway in macrophages. Myeloid-derived suppressor cells (MDSCs), another immunosuppressive population, are also expanded/functionally enhanced in high lactate conditions [11]. Dendritic cells (DCs), which are vital for antigen presentation and T cell priming, lose their function in an acidic, lactate-rich milieu; they show reduced antigen presentation and IL-12 production, thereby failing to activate CTLs. Lactate can even directly inhibit DC maturation and motility [11].

Moreover, regulatory T cells (Tregs), which dampen immune responses, are relatively more tolerant of lactate. Tumor-infiltrating Tregs use lactate as a fuel to support their suppressive functions; they express high levels of MCT1 to import lactate [53,54]. Lactate essentially feeds Tregs and helps them to proliferate in the tumor, outcompeting effector T cells that are incapacitated by the low pH. This differential effect fortifies the immunosuppressive shield around the tumor [53,55]. Lactate also boosts Treg expression of the checkpoint receptor PD-1, further inhibiting anti-tumor T cell activity [56,57]. All these factors—fewer active CTLs and NK cells as well as more TAMs, MDSCs, and Tregs—converge to create an immune-privileged environment for the cancer. Empirically, high intratumoral lactate levels have been associated with poor patient outcomes and resistance to immunotherapies [34,56,57].

**Lactate as an Immunomodulator and Potential Activator (Systemic Context):** In striking contrast to above, new evidence suggests that lactate can also have *pro*-immune effects under certain conditions. A study in 2022 showed that providing lactate systemically can rejuvenate T cells and enhance their cancer-fighting ability [58]. Lactate-treated tumors had greater infiltration of CD8+ T cells, and these T cells displayed a “stem-like” phenotype with less exhaustion [58]. Essentially, lactate exposure kept T cells in a naiver or memory-like state (higher stemness), which is favorable for sustained anti-tumor immunity. When lactate therapy was combined with checkpoint inhibitor immunotherapy, tumors completely regressed in roughly half of the mice. This synergistic effect indicates that lactate was bolstering the immune response unleashed by the checkpoint blockade [58,59].

This seemingly paradoxical result can be understood by the context: lactate was given without an associated drop in pH. In buffered form, lactate can be taken up by T cells and used in their metabolism. T cells (especially memory T cells) can utilize lactate as a substrate in their oxidative metabolism when glucose is scarce, aiding their survival and function [60]. Additionally, lactate may trigger certain signaling cascades; for instance, lactate can stabilize HIF-1α in T cells, which, in some contexts, promotes their adaptation to hypoxia and longevity [50]. The concept of lactate enhancing T cell stemness is particularly intriguing. It suggests that a moderate lactate environment might prevent T cells from differentiating into short-lived, terminal effectors and instead maintain a pool of long-lasting, proliferative T cells that can continue attacking the tumor [26]. This finding has opened up interest in whether exercise-induced lactate could partially mimic such an effect in patients.

Exercise is known to transiently mobilize immune cells [61]. During aerobic exercise in healthy participants, stress hormones (e.g., epinephrin) temporarily boost the circulation of NK cells and T cells. These cells home in to tissues 1–3 h post exercise [45,62,63]. Some studies using mouse models have shown that chronic exercise can increase immune infiltration into tumors and slow growth [64,65,66]; for example, voluntary wheel running in mice reduced melanoma growth via epinephrin-mediated NK cell mobilization to the tumor [67]. Lactate was not the focus of those studies but it is part of the exercise milieu. An interesting speculation is that lactate released by exercising muscles might act as a chemoattractant or activation signal for certain immune cells or improve their metabolic fitness [62]. In vivo, chronic exercise causes a complex milieu change, such as increased blood flow, mild hypoxia in muscles, and the release of cytokines like IL-6, which, collectively, can modulate immunity [68]. Lactate could contribute to this “exercise-induced immunomodulation” by, for instance, activating signaling pathways in immune cells or by being shuttled to lymphoid organs where immune cells reside [69,70].

The dualistic effects of lactate on immunity underline the importance of pH and concentration. Lactic acid suppresses immune cells [71], whereas lactate (as a metabolite in a physiological range with a normal pH) can support immune cell function [71,72]. Additionally, acute vs. chronic exposure matters [11]. Short-term lactate exposure, such as during exercise, is followed by a recovery period that may allow immune cells to reset and even become more effective (for example, via an increased expression of enzymes to utilize lactate or improved mitochondrial function) [71]. In this setting, blood lactate can rise to 4–10 mmol/L for a short duration (from minutes to an hour) but pH remains near normal due to effective buffering [73]. Chronic lactate exposure in a tumor affords no recovery and continuously pushes immune cells into dysfunction. The density of cells is another factor: a dense cluster of tumor cells and immune cells in a confined TME will accumulate lactate to higher levels than the same amount of lactate diluted in the bloodstream or muscles [69].

Although these immunological effects of lactate are well-documented, it is important to recognize that not all cancer types rely equally on glycolytic metabolism and the Warburg effect, as mentioned previously. This metabolic heterogeneity suggests that the immunological consequences of exercise-induced lactate may vary by tumor type. In highly glycolytic tumors, transient systemic lactate from exercise may enhance immune infiltration without worsening the acidic tumor microenvironment, while in more oxidative tumors, benefits may be primarily mediated through systemic immune modulation and improved metabolic health [16]. Thus, the Warburg dependency of each tumor may influence how exercise and lactate exposure affect cancer progression and immune engagement. Some studies reported null or weak effects of exercise in cancers that are less reliant on the Warburg effect, such as prostate [44], chronic lymphocytic leukemia [16], and renal cell carcinoma [74,75].

**Clinical Implications**

**Exercise, Lactate Modulation, and Patient Outcomes**

**Exercise Oncology Outcomes:** The consistent finding from clinical studies is that exercise is safe and beneficial for most cancer patients, including those undergoing chemotherapy or radiation [76]. Structured exercise (aerobic, resistance, or combined) improves patients’ cardiorespiratory fitness, muscle strength, fatigue levels, and quality of life. Importantly, observational studies link higher physical activity to a lower risk of cancer recurrence and cancer-specific mortality, particularly in breast and colon cancers. For example, women with breast cancer who perform moderate exercise (like brisk walking ~3–5 h per week) have significantly lower recurrence and death rates compared with sedentary patients, presumably due to improvements in metabolic and immune health. Although these studies do not measure lactate directly, they suggest that the net effect of exercise—despite transient lactate rises—is clearly positive in the oncology setting [77,78].

A key clinical question has been whether certain types or intensities of exercise could be harmful for cancer patients by exacerbating tumor metabolism. High-intensity interval training (HIIT) and vigorous anaerobic exercise produce the highest lactate levels; however, trials implementing such exercises in survivors have generally shown no evidence of tumor promotion [44]. There are anecdotal concerns that extreme endurance events could cause temporary immunosuppression (the “open window” theory, where infection risk is elevated) [79]. Yet, in the long run, endurance athletes have a robust immune function and reduced systemic inflammation. Translating this into cancer, a single bout of exhaustive exercise might transiently increase circulating growth factors or stress hormones but it could also mobilize immune cells into the circulation and tumors [80]. Clinicians, therefore, encourage regular moderate exercise (i.e., 55 to 70% of the heart rate maximum) for patients and survivors, and even supervised vigorous exercise when appropriate, as the benefits outweigh the theoretical risks. In line with this, there is no epidemiological evidence to indicate that high-intensity lifetime exercise (with frequent lactate surges) predisposes to cancer; in fact, it is associated with a reduced cancer risk [81].

**Lactate Levels as Biomarkers:** In oncology practice, lactate is sometimes measured as a biomarker of the tumor burden or aggressiveness [82,83]. For instance, serum lactate dehydrogenase (LDH), the enzyme that interconverts pyruvate and lactate, is often elevated in patients with metastatic disease and correlates with a worse prognosis in cancers like melanoma and lymphoma. High LDH (and, by extension, high lactate production) reflects an extensive Warburg effect in the body [41,84]. However, exercise can confound these measurements if undertaken before blood draws; an acute workout might temporarily elevate systemic lactate and LDH [85,86]. Patients are usually advised not to exercise strenuously immediately before blood tests to avoid such interference [87]. Conversely, improving a patient’s fitness might lower resting lactate levels and LDH over time, potentially reflecting a less inflammatory and better-oxygenated tissue environment [82,83].

**Exercise Prescription and Timing:** From a practical clinical standpoint, exercise is now often prescribed as part of comprehensive cancer care. Oncologists and physiologists are refining exercise prescriptions to maximize anti-cancer benefits [88]. One emerging idea is timing exercise with treatment cycles. Human studies showed that mild exercise before chemotherapy could increase tumor blood flow, improving drug delivery, and could also raise immune cell circulation that could attack tumor cells injured by chemotherapy [89,90]. Similarly, exercising on the day of immunotherapy infusion might theoretically enhance the treatment’s efficacy by acutely elevating levels of cytokines and immune cells [91]. As for lactate, moderate-intensity aerobic sessions (which keep lactate levels relatively low) are easily sustainable for patients and improve aerobic metabolism over time, potentially countering the Warburg effect in normal tissues [88,89]. High-intensity sessions with an intensity higher than 70% of the maximum heart rate (spiking lactate) might be used more sparingly or when a patient is fitter, perhaps to harness some of the lactate-induced immune effects [90]. Clinical trials are currently underway testing different exercise intensities for patients to see if outcomes differ; so far, none has reported any safety issue regarding tumor growth [92].

In advanced cancer patients with a heavy disease burden, caution is warranted as their baseline lactate may already be elevated due to tumor metabolism and possibly cachexia [93]. These patients might experience fatigue or breathlessness with minimal exertion (partly from anemia or metastases), so exercise intensity should be individually tailored [43,94]. Any lactate rise from exercise in such cases is likely to be small compared with the tumor’s own output, so the concern is more about patient comfort and safety than feeding the tumor. Importantly, even light exercise, with an intensity less than 50% of the maximum heart rate, like slow walking or resistance band exercises, can confer benefits in mood and function for advanced patients without meaningfully affecting lactate [42,43,94].

The last point to consider is that measuring lactate pre/post exercise sessions may help calibrate intensity, targeting ~2–4 mmol/L for aerobic benefits or 6–8 mmol/L in fitter patients for HIIT-like immune stimulation (without exceeding fatigue thresholds).

**Figure 1:**
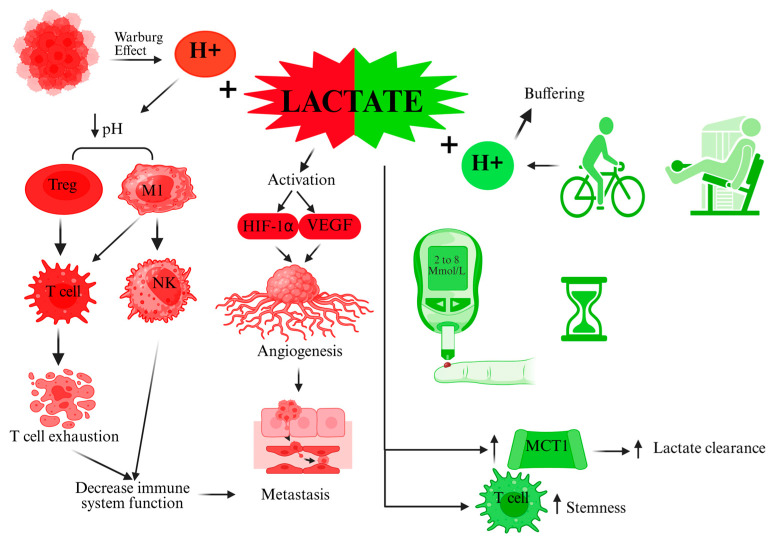
Lactate role in cancer metabolism: all about the context. Created using BioRender (Web version). Santos lozano, A. (2025) https://BioRender.com/s6s4gei (accessed on 15 March 2025).

In summary, the clinical message is that exercise is an ally for cancer patients. The lactate produced by exercise does not negate these benefits; if anything, lactate’s systemic effects might contribute to the positive immune and metabolic changes seen in exercised patients. At the same time, therapies that reduce tumor lactate production or action are being pursued to break the vicious cycle of immune suppression in the TME. By managing lactate—encouraging it in the right context and blocking it in the wrong context—we can tilt the balance in favor of the patient (see Figure 1).

**Future Directions**

Considering lactate role as context-dependent, could open a new window for future research and therapy development.

**1. Optimizing Exercise Regimens:** Prospective studies should focus on determining the optimal “dose” and type of exercise for cancer patients. Which one has the highest anticancer effects: moderate continuous or high-intensity intervals training? Does exercise induced acute lactate elevation bring benefits beyond improved fitness? Studies using the remote sensing of tumors during exercise (e.g., functional imaging) could reveal if exercise acutely alters tumor perfusion, oxygenation, or metabolism (lactate levels) in real time. Additionally, exercise individualization—adjusting intensity to a patient’s fitness and tumor characteristics—is an important point to consider. For instance, exercise program that improves aerobic metabolism could be more beneficial for a patient with a higher glycolytic tumor compared with healthy cells due to the possibility of countering Warburg effect).

**2. Unraveling Lactylation and Epigenetics:** Histone lactylation, a recently discovered mechanism, and its scope in cancer and immune cells remains unknown. Future studies should focus on the difference between exercise vs. tumor induced lactate in lactylation patterns genome-wide. Could exercise reduce harmful lactylation in TAMs or, conversely, does it increase beneficial lactylation in some contexts?

## 4. Conclusions

Lactate’s reputation for cancer has evolved from being a passive end-product of the Warburg effect to a dynamic regulator that can either promote or hinder cancer progression. This narrative review highlights that exercise-induced lactate and tumor-derived lactate are not equivalent; the former occurs in a controlled systemic milieu and can engender positive metabolic and immune effects, whereas the latter accumulates in a poorly perfused microenvironment to the tumor’s advantage. Lactate is a “foe” when its concentration is continuously high feeding the carcinogenic cells, stimulating angiogenesis, invasiveness, and immune escape by creating an acidic, immunosuppressive niche. However, it can be a “friend” when present transiently or/and systemically as it can act as a fuel for normal cells and even rejuvenate exhausted T cells, contributing to the anti-tumor immune response. Indeed, the lactate effect is “all about the context” and factors such as concentration, pH, timing, and co-signals determine lactate’s role in cancer biology.

Based on provided interpretations, we highly encourage cancer patients to participate in physical activity and confirm that fears of exercise “feeding the tumor” are largely unfounded. On the contrary, by improving cardiovascular function and immune competence, exercise may help patients better withstand treatments and potentially inhibit tumor growth, even if the mechanistic link to lactate is indirect.

## Figures and Tables

**Table 1:** Blood lactate concentration for cancer patients in different contexts.

Context	Estimated Blood Lactate Levels (mmol/L)	Cancer Type	Reference	Note
Rest (sedentary cancer patient)	1.5	Advanced solid tumors (general)	[41]	May be elevated due to tumor metabolism or cachexia
Low-intensity walking (<40% HRmax)	2	Breast, colorectal, and prostate	[42]	Mild exertion; lactate slightly increases with fatigue
Moderate aerobic (55–70% HRmax)	3	Breast, lung, and colorectal	[43]	Safe and well-tolerated; modest transient lactate rise
Supervised HIIT (85–90% HRmax)	5	Prostate, lung, and lymphoma (survivors)	[44]	Requires high fitness or supervision; buffered rise
Maximal effort test (e.g., CPET)	6	Breast and lung (during CPET or trials)	[45]	Rarely performed unless in a trial; peak still lower than healthy due to lower capacity
